# Delayed Protein Changes During Seed Germination

**DOI:** 10.3389/fpls.2021.735719

**Published:** 2021-09-15

**Authors:** Bing Bai, Niels van der Horst, Jan H. Cordewener, Antoine H. P. America, Harm Nijveen, Leónie Bentsink

**Affiliations:** ^1^Wageningen Seed Science Centre, Laboratory of Plant Physiology, Wageningen University, Wageningen, Netherlands; ^2^Bioinformatics Group, Wageningen University, Wageningen, Netherlands; ^3^BU Bioscience, Wageningen Plant Research, Wageningen, Netherlands; ^4^Centre for BioSystems Genomics, Wageningen, Netherlands; ^5^Netherlands Proteomics Centre, Utrecht, Netherlands

**Keywords:** ribosome, proteome, translation delay, seed germination, translatome, *Arabidopsis* thaliana, polysome profiling

## Abstract

Over the past decade, ample transcriptome data have been generated at different stages during seed germination; however, far less is known about protein synthesis during this important physiological process. Generally, the correlation between transcript levels and protein abundance is low, which strongly limits the use of transcriptome data to accurately estimate protein expression. Polysomal profiling has emerged as a tool to identify mRNAs that are actively translated. The association of the mRNA to the polysome, also referred to as translatome, provides a proxy for mRNA translation. In this study, the correlation between the changes in total mRNA, polysome-associated mRNA, and protein levels across seed germination was investigated. The direct correlation between polysomal mRNA and protein abundance at a single time-point during seed germination is low. However, once the polysomal mRNA of a time-point is compared to the proteome of the next time-point, the correlation is much higher. 35% of the investigated proteome has delayed changes at the protein level. Genes have been classified based on their delayed protein changes, and specific motifs in these genes have been identified. Moreover, mRNA and protein stability and mRNA length have been found as important predictors for changes in protein abundance. In conclusion, polysome association and/or dissociation predicts future changes in protein abundance in germinating seeds.

## Introduction

The central dogma in molecular biology is that a gene is transcribed into a mRNA, which is further translated into a protein. Data produced by the high-throughput techniques, such as next-generation sequencing and mass spectrometry, have shown that there is a lack of correlation between mRNA levels and protein abundance levels (Chen et al., 2002; Nie et al., 2006; Haider and Pal, 2013). This lack of correlation might partly be hampered by the low throughput of protein analysis compared to the high-throughput transcriptome analysis. In addition, cellular processes, such as posttranscriptional and translational regulation as well as different half-lives for mRNAs and proteins, limit correlation between the mRNA and protein levels (Varshavsky, 1996; Grolleau et al., 2002; Becker et al., 2018). Multiple studies have shown that protein abundance levels are better explained by ribosome-associated mRNA (translatome) measurements than by transcriptome data in a wide variety of organisms. Based on this, the translatome is proposed as a better proxy for predicting proteomic changes (Beyer et al., 2004; Lu et al., 2007; Smircich et al., 2015). In this study, germinating seeds were used as a system to identify whether indeed the translatome can be used to predict protein level changes.

Seed germination is an important trait that secures seedling establishment and thus the next generation of a plant. Plant seeds accumulate myriads of mRNAs during seed maturation, and these mRNAs are translated during early seed imbibition (Rajjou et al., 2004). This temporal separation of transcription and translation is similar to translational events during embryo development in other organisms (Kronja et al., 2014). Moreover, it has been shown that the translation of these seed stored mRNAs is essential for seed germination (Suzuki and Minamikawa, 1985; Beltran-Pena et al., 1995; Bai et al., 2020). Seed germination is a triphasic process triggered by the uptake of water, referred to as imbibition. Phase I involves water uptake during which *de novo* transcription is not essential (Rajjou et al., 2004). This phase ends when the water uptake reaches a plateau. In phase II, multiple essential cellular and biochemical processes, including protein synthesis, are activated. Phase III is the phase of *sensu stricto* germination ending with the embryonic root emergence (Bradford, 1990). Previously, we have shown substantial translational regulation of mRNAs at specific developmental stages during seed germination. Thousands of mRNAs showed distinct patterns of expression between the total mRNA and polysomal mRNA pool. This indicates that transcribed mRNAs are not always bound by ribosomes and directed for protein synthesis (Bai et al., 2017). Polysome profiling is tool to characterize the polysome-associated mRNAs (translatome; Mustroph et al., 2009, Pringle et al., 2019). The comparison of the polysomal associated mRNAs to the total mRNA on a genome-wide scale hints at the translational efficiency of these mRNAs (Branco-Price et al., 2005; Gamm et al., 2014; Bai et al., 2017, 2018). However, it is not known if polysomal mRNA changes can predict the protein abundance during the seed germination. Here, we combine our previous study in which we have identified polysome-associated mRNAs during seed germination with protein abundance obtained from the same samples. Therefore, disturbance by repeated sampling variation can be excluded. We reveal a global delay in protein-level changes and identified potential sequence features that might contribute to this delay in protein-level changes. Specific motifs identified on the mRNAs for which the protein-level changes are delayed suggest a role for RNA-binding proteins (RBPs) in mediating a delay in protein translation or degradation.

## Materials and Methods

### Plant Material Sampling and Total Protein Extraction

Maturing *Arabidopsis thaliana* seeds (accession Columbia-0) were grown in three biological replicates with 50 plants each. Plants were grown on 4×4cm Rockwool blocks irrigated with standard nutrient solution as (He et al., 2014) in a growth chamber at 20/18°C (day/night) under a 16-h day/8-h night photoperiod of artificial light (150μmolm^−2^s^−1^) and 70% relative humidity. Seeds at specific physiological states during germination were harvested and frozen in liquid nitrogen, freeze-dried, and stored at −80°C until further analyzed. Protein extraction, purification, chromatography and mass spectrometry and database search for protein identification, and quantitative analysis are included in Supplementary Methods.

### Data Retrieval and Statistical Analysis

The microarray data of total mRNA and polysomal mRNA were retrieved from the Gene Expression Omnibus repository^1^ accession number GSE65780 (Bai et al., 2017). Log2-fold transformed microarray intensity data for the transcriptome/translatome and Log2 LFQ abundance signal for the proteomic data were used for the correlation analysis. Furthermore, sequence data [5'UTR, 3'UTR, and CDS (coding DNA sequence)] and predicted subcellular location were retrieved from the TAIR10 database from The Arabidopsis Information Resource (TAIR; Berardini et al., 2015). To identify any bias in the data, the total mRNA and polysomal mRNA Log2 intensity data were retrieved for the genes for which protein abundance is available from the current study. The expression for the subset which the protein data are available from current study is compared to the whole dataset, including all expressed genes on mRNA and polysomal mRNA level in the seeds (19,781 genes). The Wilcoxon Rank Sum test is used to test whether there is a significant difference between the groups. The predicted subcellular location was retrieved from the TAIR10 database, and the subcellular enrichment was calculated for the complete gene set and the subset for which protein expression was available. For correlation analysis, the Shapiro test was used to calculate whether the mRNA, polysomal mRNA, and protein abundance data are normally distributed. Spearmen correlation analysis was used to calculate the correlation coefficient. To calculate the differential expression between the time-points on polysome-associated mRNA and protein abundance level, a linear model based on empirical Bayes methods was used (Smyth, 2004) and applied by log2-fold change > 1 and *p*-value < 0.05 and adjusted by the false discovery rate calculated using the Benjamini and Hochberg (1995). The statistical analyses in this study were performed using R version 3.6.1^2^ in RStudio.^3^

### Subcellular Localization Classification and GO Enrichment Analysis

Subcellular localization data from TAIR10 were used to classify protein for their localization. The ratio of the protein from each subcellular location was calculated based on the number of proteins identified from each location in the proteomic dataset and the total number of proteins identified. ClusterProfiler (Yu et al., 2012) was used for GO enrichment analysis with total gene identified from proteomic dataset (1,438 genes) as background. GO terms were considered significant if the *p*-value < 0.05 adjusted for the false discovery rate calculated using the Benjamini and Hochberg (1995) method.

### Mass Spectrometry and Proteomic Analysis

Raw lysate from the same sample extract as performed for total mRNA and polysomal mRNA analysis were used for total protein extraction (Bai et al., 2017). The detailed protein extraction, chromatography and mass spectrometry, and also the proteomic analysis are described in the Supplementary Information.

### mRNA and Protein Sequence Analysis

mRNA and protein decay rata data were retrieved from Narsai et al. (2007) and Li et al. (2017). The distributions of sequence lengths of each compared group were evaluated separately for CDS, 5' untranslated region (5'UTR), 3'UTR, and full transcript as compared to the total identified protein coding genes from proteomic analysis as background. Given the non-normality of the distributions of values, a Wilcoxon signed-rank test was adopted for all statistical comparisons (median as the test statistic).

### Motif Analysis

DNA motif analyses were performed using the MEME suite (Bailey et al., 2009), for full transcript, 5'UTR, CDS, and 3'UTR sequences, extracted from the TAIR10 database.^4^ The minimum and maximum motif widths were set to five and 12, respectively. If a gene had multiple transcripts, only the TAIR10 representative splice form (first splicing form in the database) was used. Background dinucleotide frequencies were provided separately for each sequence type (including 5'UTR, CDS, cDNA, and 3'UTR). FIMO (Bailey et al., 2009) was used to test motif specificity, motifs with a FIMO adjusted *p*-value lower than 0.001 were considered as significant hits. Obtained motif counts from FIMO (Version 5.3.0) were used to compute the enrichment *p*-value for the identified genes using all mRNAs expressed in the experiment as background by means of a one-tailed Fisher’s exact test, performed with a custom script and the R software package^5^ where *p*-value < 0.05 was considered to be significant. FIMO is used for motif scanning to identify the gene transcripts at the region where the motif is identified. The identified motifs and RNA sequence from the regulated gene sets (including 5'UTR, CDS, cDNA, and 3'UTR) were used as input for the sequence scan. Value of *p*<0.0001was used as cutoff to filter the matched sequences.

## Results and Discussion

### Protein Expression Data Are Enriched for Highly Expressed Genes

**Figure 1:**
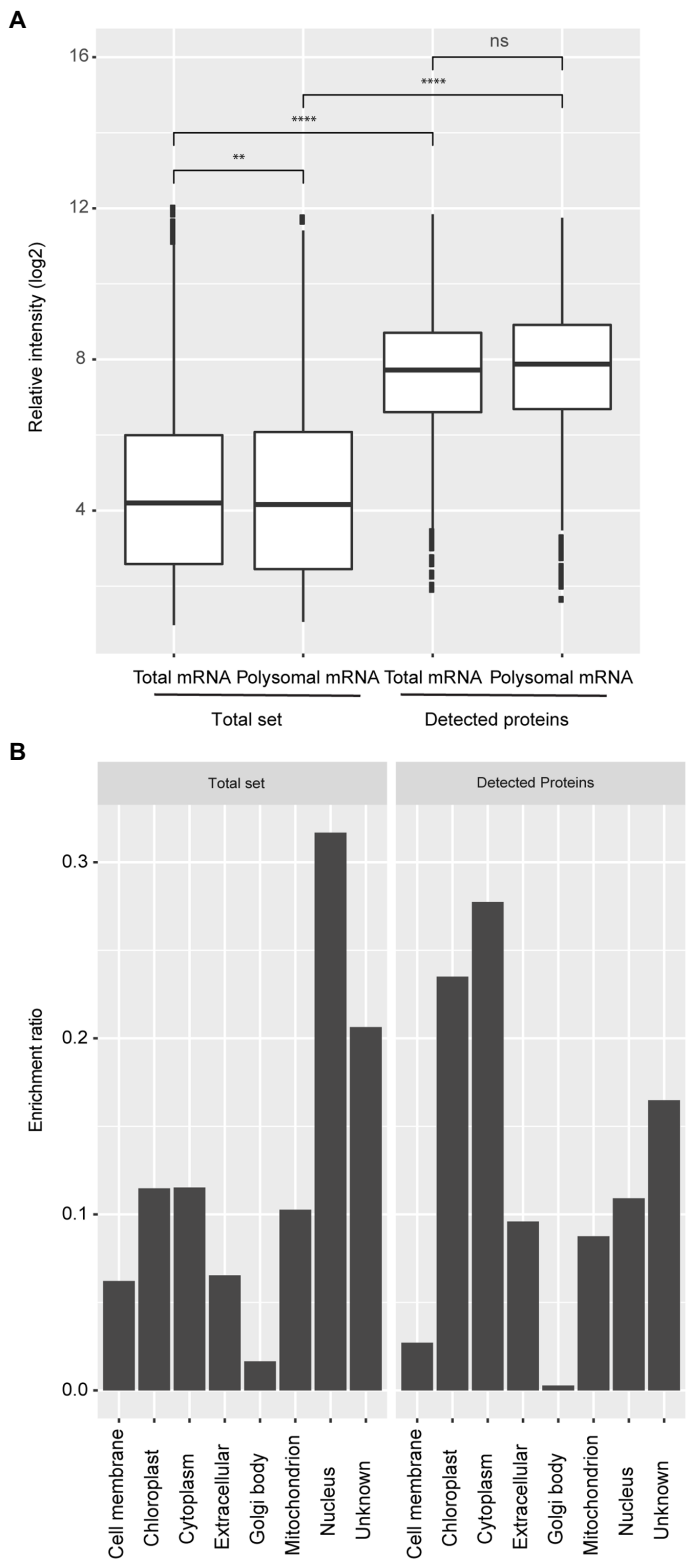
Expression bias of detected protein coding genes and their predicted subcellular localization. **(A)** Boxplots representing the expression level of total and polysomal mRNA identified in the total set of genes that are expressed in seeds (Total set) and total and polysomal mRNA of the subset for which proteins have been identified (Detected proteins). The boxplots are based on the data from five time-points with three biological replicates (ns; *p*>0.05; ^**^p<0.01; ^***^p<0.001; ^****^p<0.0001; Wilcoxon rank-sum test). **(B)** Classification of subcellular locations of the genes for the whole set (Total set) and subset (Detected proteins). The ratio was calculated by dividing the number of genes/proteins from each subcellular location and the total number of genes/proteins identified from each dataset.

Protein levels at five distinct physiological stages as Bai et al. (2017) during seed germination (dry, 6, 26, 48, and 72 hours after imbibition; HAI) were measured by Liquid Chromatography-Mass Spectrometry (LC-MS). Proteins were precipitated using chloroform/methanol instead of the canonical acetone precipitation. This resulted in a high protein recovery and an optimal solubility which is requested for the LC-MS analysis. An additional acetone precipitation gained only 5.8% of total protein; based on this result, it was decided to stick to the chloroform/methanol precipitation for further analyses (Supplementary Table 2, Supplementary Information). In total, 1,469 proteins were identified. To be marked as identified proteins had to be present in all three biological replicates in at least one of the five physiological stages (Supplementary Table 1). The number of identified proteins is much lower than the number of transcripts identified in transcriptome and polysome analyses that were performed on exact the same samples, which resulted in 19,781 expressed genes (Bai et al., 2017). Comparative analyses revealed that the proteins identified represent genes that are significantly higher expressed at both the mRNA and polysomal mRNA level compared to the total set of genes expressed in germinating seeds (Wilcoxon Rank Sum test, *p*-value < 0.0001; Figure 1A; Supplementary Figure S1). There was a bias in the predicted subcellular locations for the detected proteins. Genes localized in the chloroplast and cytoplasm were over-represented, while nucleus-localized genes were under-represented (Figure 1B). This bias is likely the result of the applied protein isolation method and the detection limit of the LC-MS. Proteins localized in the nucleus, such as transcription factors and signaling proteins, might therefore be excluded from these analyses. Abundant proteins dominate the LC-MS identification, limiting the detection of low abundant proteins. Moreover, in seeds, the most abundant proteins are located in the cytosol (Mamontova et al., 2019). Also, the protein isolation buffer may be more efficient for purifying hydrophilic proteins. Despite the possible bias in the proteome subset (1,438 proteins), these data were used to compare protein abundance, polysomal, and total mRNA levels. Comparing the different levels of gene regulation is currently largely unexplored in plants; however, this dataset on seed germination is unique in that it allows such a systematic comparison.

### Polysomal mRNA and Protein Abundance Display a Delay in Protein-Level Changes

**Figure 2:**
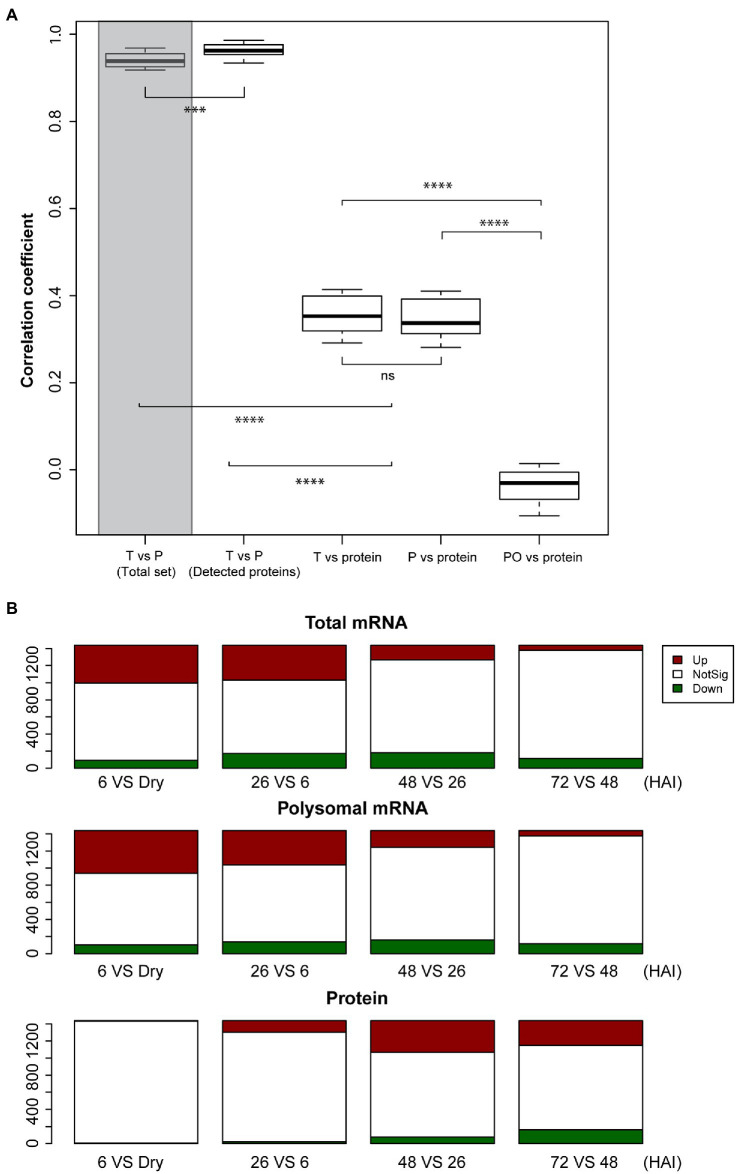
Lack of correlation between total mRNA, polysomal mRNA, and protein abundance during seed germination. **(A)** Distribution of Spearman correlation coefficients (five time-points and three biological replicates) between total mRNA (T) and polysomal mRNA (P) for the total set of identified mRNAs (Total set; shaded bar) and the mRNAs with identified protein (Detected proteins), total mRNA and protein, polysomal mRNA, and protein as well as the polysomal occupancy (PO) and protein (ns; *p*>0.05; ^**^p<0.01; ^***^p<0.001; ^****^p<0.0001; Wilcoxon rank-sum test). **(B)** Differential expressed genes in total mRNA, polysomal mRNA, and protein during seed germination following hours after imbibition (HAI). The genes upregulated (Up, red) and downregulated (Down, green) are displayed in comparison with the non-significant (NotSig, white) changed genes/proteins.

To investigate the correlation between transcriptome, translatome, and proteome expression, spearman correlation analyses were performed. A strong correlation was found between the total and polysomal mRNA in the proteome subset (*r*=0.96); this correlation was significantly higher than that in the whole dataset (27,826 genes on the microarray; *r*=0.94, Wilcoxon Rank Sum test, *p*-value < 0.001; Figure 2A). This is consistent with the high correlation between the transcriptome and translatome observed in mice tissue (Reid et al., 2017). In contrast, the correlation between mRNA and protein, and polysomal mRNA and protein is both relatively low (*r*=0.36 and *r*=0.35, respectively; Figure 2A). This low level of correlation may have different explanations: (1) It might be bias caused by the fact that only the high expressed subset of the total number of genes that are expressed in seeds was taken into account, (2) there can be a delay in translation for translationally regulated proteins before translated proteins accumulate to a level that will be detected by proteomics, (3) it is also possible that translation in seeds occurs by single ribosomes (monosome) as well and not only by polysomes as is often thought (Bai et al., 2020), or (4) not all the polysome-associated mRNA are eventually translated into proteins due to, e.g., ribosome stalling (Rachter and Coller, 2015). Monosomes have been excluded in our analyses. For specific tissues, it has been shown that translation occurs by monosomes, *e.g*., in the tiny synaptic compartment of rodent. mRNA is translated by monosomes rather than by polysomes, likely the result of the limited space in these neuron cells (Biever et al., 2020). Also, ribosomes can bind to mRNAs without translating, as was shown by the repression of translation initiation of the ribosome-associated *HAC1* mRNA due to lack of splicing in *Saccharomyces cerevisiae* (Kuhn et al., 2001). The correlation between the polysome occupancy (as proxy for translational efficiency) and protein abundance is also low (Figure 2A), revealing an uncoupling between polysome association and protein abundance during seed germination. This contrasts with the study by Beyer et al. (2004). These authors showed a slightly higher correlation between translational efficiency (represented by ribosome density) and total protein abundance than between total mRNA and total protein abundance. This discrepancy is likely caused by the translational status of the two different systems. In yeast, translation is sustained in a constantly active state, while in seed, translation is activated during germination after being completely inactive in the dry stage (Bai et al., 2017).

**Figure 3:**
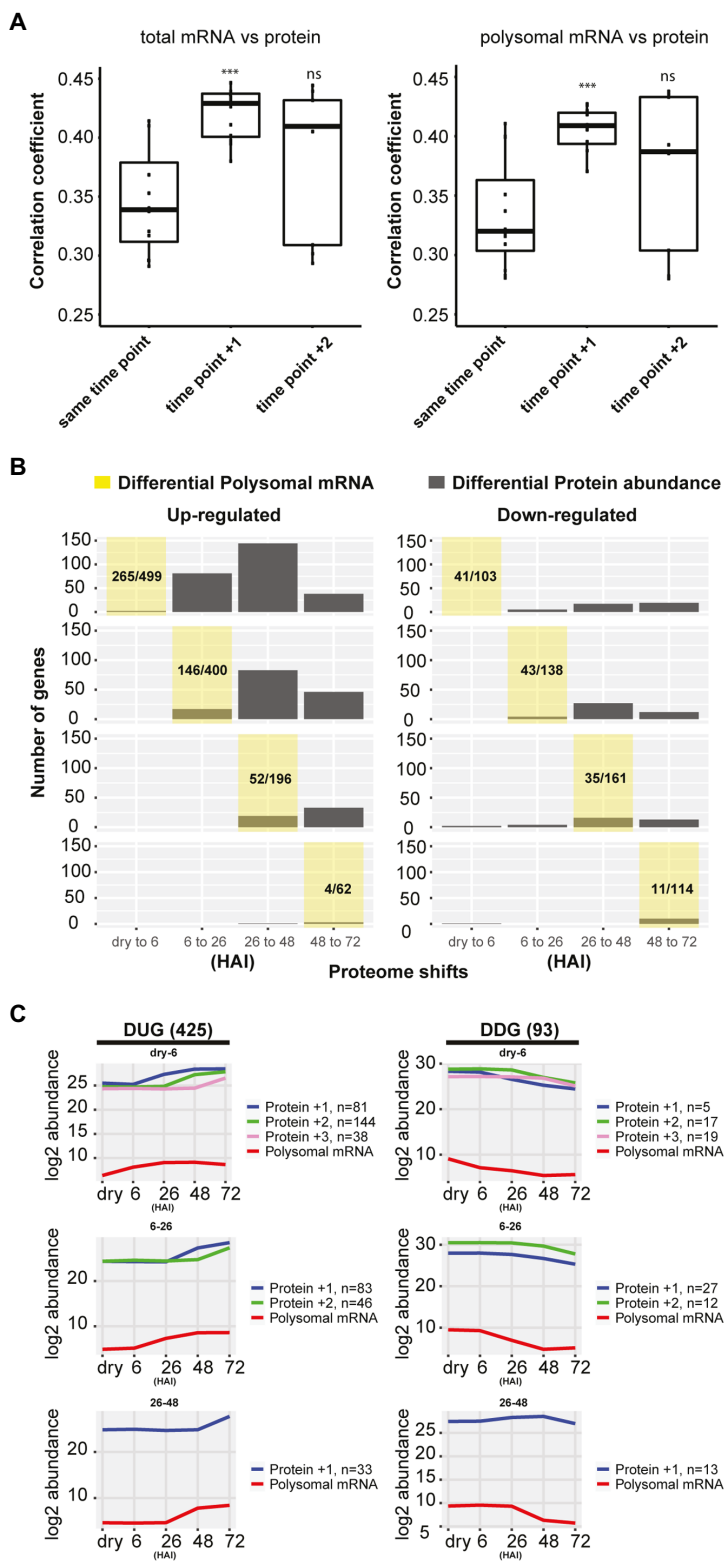
Delay in protein synthesis during seed germination. **(A)** Correlation analysis between the total mRNA, polysomal mRNA level, and the protein abundance of the next time-points. Time-points +1 and +2 for the polysomal mRNA are used for the correlation with the protein abundance (^***^p<0.001; Wilcoxon rank-sum test). **(B)** Delay in protein abundance changes compared to changes in polysome-associated mRNAs comparing two consecutive hours after imbibition (HAI) time-points. The yellow shaded area represents the germination time-point under investigation and indicates when the polysomal mRNAs are for the first time up-/downregulated. The corresponding distribution of protein changes of these genes changed in polysomal mRNA at the yellow shaded time-point is indicated in grey. The proportions of up- or downregulated polysomal mRNA for the detected proteins (sum of grey bars) compared to the total number of differentially regulated polysomal mRNA at each time-point are shown. **(C)** The average abundance profiles of the polysome-associated mRNA and proteins of the delayed response genes at different HAI during seed germination. At the left, the Delayed Response Upregulated Genes (DUGs) are indicated, and at the right, the Delayed Response Downregulated Genes (DDGs). The polysome mRNA is plotted at each consecutive time-point, and the corresponding protein is plotted according to the first time they are up-/downregulated indicated by +1/+2/+3 shift for the detected time-point. The gene/protein changes in each category are shown.

Possible delays in protein-level changes were investigated by comparing the total mRNA, the polysomal mRNA, and the total protein abundance at each germination time-point to its preceding time-point (Figure 2B). Differential expression analysis at total mRNA and polysomal mRNA level identified substantially more changes during the early time-points (dry, 6 and 26 HAI) compared to the later time-points (Figure 2B; Supplementary Table 3). This pattern was reversed at the protein level, where only a low number of differentially abundant proteins were identified in the early time-points and later time-points showed larger changes. Two tests were performed to investigate whether the low correlation between the polysome and the proteome could be the result of a delay of changes in protein level. First, the total mRNA and polysome mRNA at a time-point were compared to the protein abundance at the next time-point (i.e., per mRNA dry seeds vs. protein 6 HAI). This resulted in a significantly enhanced correlation (*p*-value < 0.001, from average 0.35–0.41; Figure 3A). Then, the time-point at which the gene expression changed for the first time at the polysomal level (both up and down) was compared to its protein abundance at all time-points. Similar significant correlation enhancement was identified (*p*-value < 0.001, from average 0.33–0.40). Both tests revealed that the up- and downregulation of (polysomal) mRNAs preceded the change at the protein abundance level, indicating a delay in either protein accumulation/synthesis or degradation. Since we do not know the reason for the delay, we refer to “delay in protein level changes” this article. The correlations between changes at the total mRNA and protein level and the polysomal mRNA and the protein levels were similar (Figure 3A) and indicate that the delay occurs from the polysome association to the protein synthesis.

### Unique Functions of the Delayed Response Genes

To further characterize the delayed response genes, two gene sets were created: one containing all Delayed Response Upregulated Genes (DUGs) and the other containing the Delayed Response Downregulated Genes (DDGs), 425 and 93 genes in each set, respectively (Figure 3C; Supplementary Table 4). Two public data sets were used to validate the delayed protein changes in the DUG set; these include a high-density transcriptome time-course and neosynthesized protein during seed germination (Dekkers et al., 2013; Galland et al., 2014). Six genes that were detected as *de novo* synthesized proteins were also identified in the current DUG set, and thus, their RNA profile and neosynthesized protein synthesis dynamics could be compared. The peak of protein synthesis for these six delayed genes lagged behind the peak of their corresponding mRNA signal during germination (Supplementary Figure S2). This development-dependent delay is far beyond the sequential time interval during mRNA transcription and protein translation, which normally occurs within seconds or minutes (Boehlke and Friesen, 1975; Ingolia et al., 2011). Therefore, the delay between changes at the polysomal mRNA level and actual changes in protein abundance suggests a temporal regulation between polysome association and protein synthesis/degradation. Such a phenomenon is not new to seeds. Previously, it has been reported that there is a delay between the transcription and translation of some Late Embryogenesis Abundant proteins (Galau et al., 1987, 1991; Espelund et al., 1992; Chatelain et al., 2012; Verdier et al., 2013). It has been suggested that this posttranscriptional regulation during seed maturation is linked to the plasticity of seed tissues to respond to fluctuations in the environment and as such contribute to a better seed storability (Verdier et al., 2013). Such a delay of translation also occurs during seed germination. The GO enrichment analysis showed that the DUG set was enriched for translation, ribosome biogenesis, photosynthesis, and plastid organization (FDR<0.05; Supplementary Table 5), genes that are essential for protein synthesis to support seed germination and photomorphogenesis during seedling establishment. The DDG set was enriched for the GO terms “response to abscisic acid” and “oxidation-reduction process” (FDR<0.05; Supplementary Table 5) and includes seed storage proteins, NAD(P)-binding proteins, glutathione transferases, and peroxygenases. The delayed reduction of these stress-related proteins would allow seeds to respond to changes in the environment, i.e., oxidation and maintain their plasticity when adverse conditions are met before germination (Nguyen et al., 2015; Sano et al., 2016). During seed germination, the most evident delay in protein translation occurred from 0 (dry) to 6 and from 6 to 26 HAI (Figure 3C), corresponding to seed germination Phases I and II (Bewley, 1997). From dry to six HAI, the translation of genes related to translation and ribosome biogenesis were delayed. This is consistent with the polysome profile during early seed germination, which indicates that ribosome biogenesis only starts after six HAI (Bai et al., 2017). Moreover, among the delayed genes from 6 to 48 HAI were those related to photosynthesis (Supplementary Table 5), indicating that although the mRNA is associated with polysomes, protein synthesis does not occur until the embryo needs to become autotrophic during seedling establishment.

Delaying translation might be an evolutionary advantage. The translation is energy consuming; therefore, the delay of the protein translation could increase the chance of survival in case the conditions do not allow the plant to fulfil its life cycle. Germination is an all or nothing event, and the decision to germination can only be made once. The delay of translation might refer to a checkpoint that needs to be passed for germination to occur. Until they have germinated, seeds are resilient to environmental stress and can even regain their desiccation tolerance and rehydrate and germinate when conditions allow this (Maia et al., 2011).

### mRNA Sequence Features and Protein Stability Wire the Delay in Protein-Level Changes

**Figure 4:**
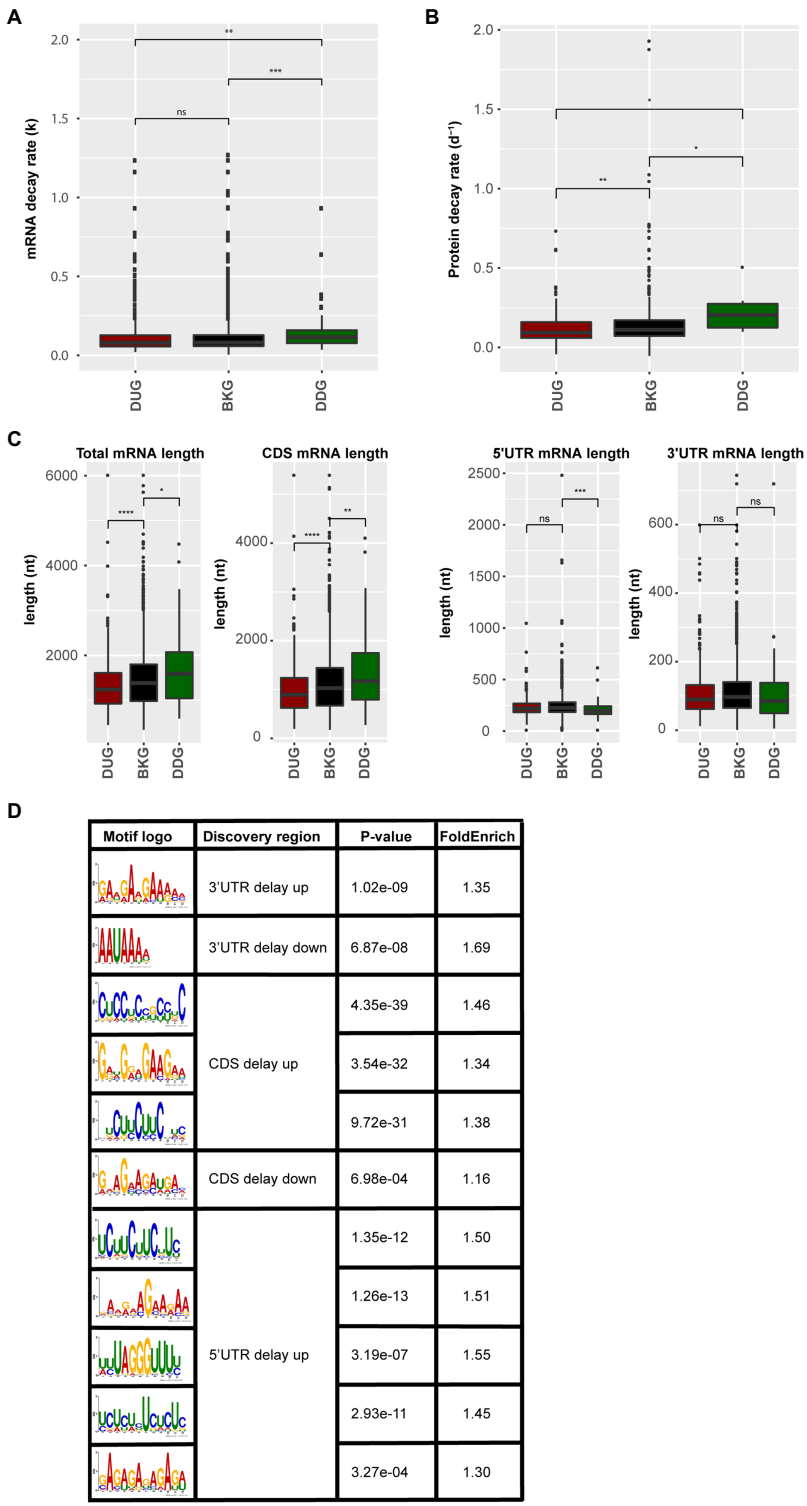
mRNA and protein sequence features for the protein delayed genes. **(A)** mRNA decay rate of the proteins that are delayed in abundance. **(B)** Protein decay rate for the delayed response genes. **(C)** mRNA sequence length including total mRNA length, length of coding sequence, 5'UTR, and 3'UTR for the delayed response genes (ns; *p*>0.05; ^*^p<0.05; ^**^p<0.01; ^***^p<0.001; ^****^p<0.0001; Wilcoxon rank-sum test). **(D)** Sequence motifs identified in the delayed response genes. Motif logo, the discovered sequence region (5'UTR, 3'UTR, and CDS), the *p*-value (Fisher test), and the fold enrichment compared to the background set (total identified protein coding genes) are shown for motifs identified in the DUG and the DDG sets.

Genes with a delayed response in protein abundance are likely under translational control. The control of mRNA translation involves the interaction between mRNA, ribosome, and associated translation factors, such as translation initiation, elongation, and termination factors. This interaction is substantially influenced by mRNA sequence features, mRNA stability, and the folding rate of the synthesized peptides (Chatelain et al., 2012; Radhakrishnan and Green, 2016; Farias-Rico et al., 2018; Waudby et al., 2019). By quantifying the mRNA decay rate and protein decay rate *in silico* for both the DUG and DDG dataset compared to the background control (Narsai et al., 2007; Li et al., 2017), it was identified that mRNA and protein stability correlated with the delay in protein changes. The mRNA stability, based on both the mRNA decay rate and mRNA half-life times, of the DDG set was significantly lower when compared to the background set (Figure 4A; Supplementary Figure S3). Similarly, the protein decay rate of the DDG set was significantly higher when considering the total set of delay genes (Figure 4B). A closer look at the time-points revealed that mRNAs high in decay rate were mainly identified from 26 to 48 HAI time-point when germination occurs (Supplementary Figures S4A–C; Supplementary Tables 6 and 7). In contrast, the DUG set mainly contained genes with the lower protein decay rate (0–26 HAI; Supplementary Figure S4C). Interestingly, the DUG set contains relatively short sequences, especially the set of genes that are expressed during early germination (0–6 HAI; Supplementary Figure S4D). The DDG set contains mainly longer genes at the same time-point (0–6 HAI; Figure 4C, Supplementary Figure S4C). These analyses suggest a role for length of the coding region (CDS), mRNA, and protein stability on the delay of the changes in protein abundance.

RNA-binding proteins are known to regulate translation by binding to specific motifs at different positions in the mRNA. To investigate a possible role for RBPs in the delay of protein changes, motif enrichment analyses were performed on the 5'UTR, 3'UTR and CDS of mRNAs in both DUG and DDG. 11 motifs were found in different regions (*p*-value <0.05), including one motif in the 5' UTR, five motifs in the 3'UTR, three motifs in the CDS of DUG set, one motif in the 5' UTR, and one motif in the CDS of DDG set (Figure 4D). Motif UCUUCUUC, identified in both the 5'''UTR and CDS of the DUG set, is a known binding site of the Glycine-rich RNA-binding protein 7 (GRB7, AT2G21660; Zhang and Mount, 2009; Meyer et al., 2017). Motif GAAGAAGA, identified from the 3'UTR of the same set, was previously identified as intronic splicing regulatory element recognized by Serine/arginine-rich splicing factor 45 (SR45, AT1G16610) and Serine/arginine-rich SC35-like splicing factor 33 (SCL33, AT1G55310), two exonic splicing enhancer with RNA binding capacity (Pertea et al., 2007; Thomas et al., 2012; Xing et al., 2015). These three RBPs are identified in the ribosome-associated mRNA fractions in seeds (Bai et al., 2020), indicating their potential role for translational regulation. The disruption of GRB7 and SR45 results in defects during seed germination and seedling growth in stress conditions, including abscisic acid hypersensitivity and retarded germination and seedling growth as a result of salt and dehydration (Cao et al., 2006; Carvalho et al., 2010). Therefore, GRB7 and SR45 are likely involved in determining the plasticity of seed germination. The regulation of mRNA translation by these RBPs likely contributes to the delay in the protein synthesis. Such a delay could reduce the rate of translation initiation and elongation, which is important for protein co-translational folding thereby ensuring the correct functionality of the encoded proteins (Zhao et al., 2019). Potential RBPs targets were identified by scanning for the sequence motif. In total, 18 mRNAs were identified to contain the GAAGAAGA motif at their 3'UTR. 54 and 66 mRNAs were identified to contain the UCUUCUUC motif in their 5'UTR and CDS, respectively (Supplementary Table 8), among which 20 and 24 were identified as GRP7 binding targets previously (Cao et al., 2006). The GAAGAAGA motif containing mRNAs was enriched for GO category of response to cold, while the UCUUCUUC motif containing mRNAs was enriched for GO categories, such as salt response, translation, and ribosome biogenesis (Supplementary Table 9). Therefore, RBPs might mediate the timing of protein translation during seed germination. Moreover, translation that is on hold or delayed might result in the formation of the so-called stress granules (SGs) or processing bodies. Earlier, we have identified SG and processing bodies proteins in association with the ribosome in seeds, which suggests that during seed germination these cellular foci are also formed (Bai et al., 2020).

## Conclusion

In this study, we have compared protein-level changes during seed germination with transcriptome and polysome changes. This unique data set allows correlation analyses between the three levels of regulation (transcriptome, translatome, and proteome) over a series of time-points. We identified a high correlation between total mRNA and polysomal mRNA levels, but only a low correlation between polysomal mRNA and protein abundance during seed germination. The low correlation is likely caused by a delay in protein translation and degradation. Multi-level correlation studies have not been performed before due to the lack of consistent sampling schemes on multiple “omic” levels. Whether the identified delay on protein level compared with polysomal mRNA level is unique for germinating seeds remains elusive since similar types of analyses have not yet been reported for other systems.

Seeds are key for plant survival, and thus, it is not surprising that the germination of seeds is strongly regulated. Seed germination can be halted at several moments and even after the embryonic root (radicle) has protruded its surrounding layers, seeds can survive desiccation (Maia et al., 2011). Earlier, we suggested a role for RBPs in the control of translation in seeds (Sajeev et al., 2019); the current study revealed some possible candidates based on the identified sequence motifs and germination phenotypes of RBPs that may recognize these motifs. In depth, studies identifying the mRNA, RBP, and ribosome interactome will allow a further investigation of the regulatory mechanisms underlying the translational control of seed germination.

## Data Availability Statement

The original contributions presented in the study are publicly available. These data can be found at ProteomeXchange with following accession: PXD027345.

## Author Contributions

BB and LB designed the experiment. BB, AA, and JC performed the proteomic analysis. BB, NH, and HN analyzed microarray and proteomic data. BB, NH, HN, and LB wrote the paper. All authors contributed to the article and approved the submitted version.

## Funding

This work was supported by The Netherlands Organization for Scientific Research and Technology Hotel Program (Grant number: 435002002) from The Netherlands Organization for Health Research and Development.

## Conflict of Interest

The authors declare that the research was conducted in the absence of any commercial or financial relationships that could be construed as a potential conflict of interest.

## Publisher’s Note

All claims expressed in this article are solely those of the authors and do not necessarily represent those of their affiliated organizations, or those of the publisher, the editors and the reviewers. Any product that may be evaluated in this article, or claim that may be made by its manufacturer, is not guaranteed or endorsed by the publisher.

## Footnotes

^1^ http://www.ncbi.nlm.nih.gov/geo/

^2^ https://www.r-project.org/

^3^ https://rstudio.com/

^4^ http://www.arabidopsis.org/

^5^ http://www.r-project.org/
