# Supplementary material for: Delayed Protein Changes During Seed Germination
Source: Front Plant Sci. 2021 Sep 15;12:735719. doi: 10.3389/fpls.2021.735719 (PMC8480309; doi:10.3389/fpls.2021.735719)
Supplement: Supplementary file 1 [file Data_Sheet_1.docx]

**Supplemental Information**

**Running Title: Protein translation during seed germination**

**Title****:** Delayed protein changes during seed germination

**Authors:** Bing Bai^1*^, Niels van der Horst^2^, Wenying Huo^1^, Jan H. Cordewener^3,4,5^, Antoine H.P. America^3,4,5^, Harm Nijveen^2^, Leónie Bentsink^1*^

^1^ Wageningen Seed Science Centre, Laboratory of Plant Physiology, Wageningen University, 6708 PB Wageningen, The Netherlands

^2^Bioinformatics Group, Wageningen University, 6700 AP Wageningen, The Netherlands

^3^BU Bioscience, Wageningen Plant Research, 6700 AA Wageningen, The Netherlands

^4^Centre for BioSystems Genomics, 6700 AB Wageningen, The Netherlands

^5^Netherlands Proteomics Centre, 3508 TB Utrecht, The Netherlands

***To whom correspondence should be addressed:** Bing Bai ([bing.bai@wur.nl](mailto:bing.bai@wur.nl)); Leónie Bentsink ([leonie.bentsink@wur.nl](mailto:leonie.bentsink@wur.nl))

Supplemental methods

Total protein extraction for proteomic analysis

Maturing *Arabidopsis thaliana* seeds (accession Columbia-0) were grown in three biological replicates with 50 plants each. Seeds at specific physiological states during germination were harvested and frozen in liquid nitrogen, freeze-dried and stored at -80 °C until further analyzed. For the isolation of total protein, 400 mg of freeze-dried tissue was extracted with 8 ml of extraction buffer (0.25 M sucrose, 400 mM Tris, pH 9.0, 200 mM KCl, 35 mM MgCl_2_, 5 mM EGTA 50 μg/mL Cycloheximide, 50 μg/mL Chloramphenicol). Chloroform/methanol rather than canonical acetone precipitation was applied in the lysate for protein precipitation based on its high protein recovery and solubility for down-stream LC-MS. In brief, 1.1 ml of methanol was added to 0.6 ml of the raw extract and mixed thoroughly. After adding 0.3 ml of chloroform, vortexing and centrifugation for 15 minutes at 16,000 g, the supernatant was carefully removed with a capillary pipette. The air-dried protein pellet was dissolved in 100 µl of 8 M urea and an aliquot was taken for protein estimation using the Qubit® protein HS Assay Kit (ThermoFisher, Waltham, USA). Between 4-15 µg of protein dissolved in 100 µl of 8 M urea was used for digestion after reduction (5 mM DTT; 30 min at 37°C) and alkylation (15 mM iodoacetamide; 30 min at RT in the dark) of the cysteines. After adding 75 µl of 0.1 M ammonium bicarbonate buffer (pH8.3), 25 µl of Trypsin/Lys-C Mix (0.02 µg/µl, Promega, Madison, USA) was added for digestion (3 hours at 37°C). After dilution with 200 µl of 0.1 M ammonium bicarbonate, 4 µl of modified trypsin (0.05 μg/μl, Promega, Madison, USA) was added for overnight incubation at 37°C. Digestion was terminated by adding 0.1% trifluoroacetic acid (TFA). The tryptic digests were cleaned by reverse phase SPE (solid phase extraction) on an Oasis HLB Elution Plate (Water). Peptides were eluted with 0.1 ml of 50% acetonitrile (ACN), 0.1% TFA and dried by vacuum centrifugation. For 2D-LC-MS analysis the peptides were dissolved in 20 μl of 0.1 M ammonium formate (pH 10) and 5 μl of the peptides were injected into the machine.

**Chromatography and mass spectrometry**

For high resolution separation of the ribosomal protein digests, a nanoAcquity 2-D UPLC system (Waters Corporation, Manchester, UK) was used employing orthogonal reverse phase separation at high and low pH, respectively. With this 2-D set up, the pool of peptides was eluted from the first dimension XBridge C18 trap column (in 20 mM ammonium formate pH 10) in two steps of 20% and 65% ACN. For the second dimension we used a BEH C18 column (75 μm ×25 cm, water UK) eluting with a 100 minutes linear gradient from 3 to 40% ACN (in 0.1% FA) at 200 nl/min. The eluting peptides were on-line injected into a Q-Exactive Plus (Thermo Scientific) mass spectrometer using a nano-electrospray source. Ionisation (2.4 kV) was performed using a stainless-steel emitter and a heated capillary temperature of 250˚C. Full MS scans were acquired over the m/z range 400-1500 with a mass resolution of 70 000 (at m/z 200). Full scan target was set 3x106 with a maximum fill time of 50 ms. The 10 most intense peaks with charge state 2-4 were fragmented in the HCD collision cell with a normalized collision energy of 28%. The mass range was set to 140-2000, and a mass resolution of 17500 (at m/z 200). The target value for fragment scans was set at 5x104, the intensity threshold was kept at 1x104 and the maximum allowed accumulation times were 100 ms. Peptide match was set to preferred, isolation window at 1.6 m/z and isotope exclusion was on. The dynamic exclusion was set to 30 s.

**Database search for protein identification and quantitative analysis**

The raw QEX LC-MS/MS data were processed with the work flow described for plant proteomics using the Maxquant (Cox and Mann, 2008; Smaczniak et al., 2012) and the resulting masses file containing all the fragment information was matched against the an in-house TAIR protein sequence database based on The Arabidopsis Information Resource (TAIR10, http://www.Arabidopsis.org). The proteinGroups.txt file generated from Maxquant was used for quantitative analysis. Intensity determination and normalization was performed by algorithm MaxLFQ (Cox et al., 2014). The LFQ value generated was used for further statistics analysis by package Perseus (Tyanova et al., 2016). A protein was identified as present if Log2 transformed LFQ value is above background for at least in the three biological replicates of at least one sample. An imputation is used to simulate the missing values based on the normal distribution with simulation with of 0.3 and shift of 1.8.

**Supplemental figures**


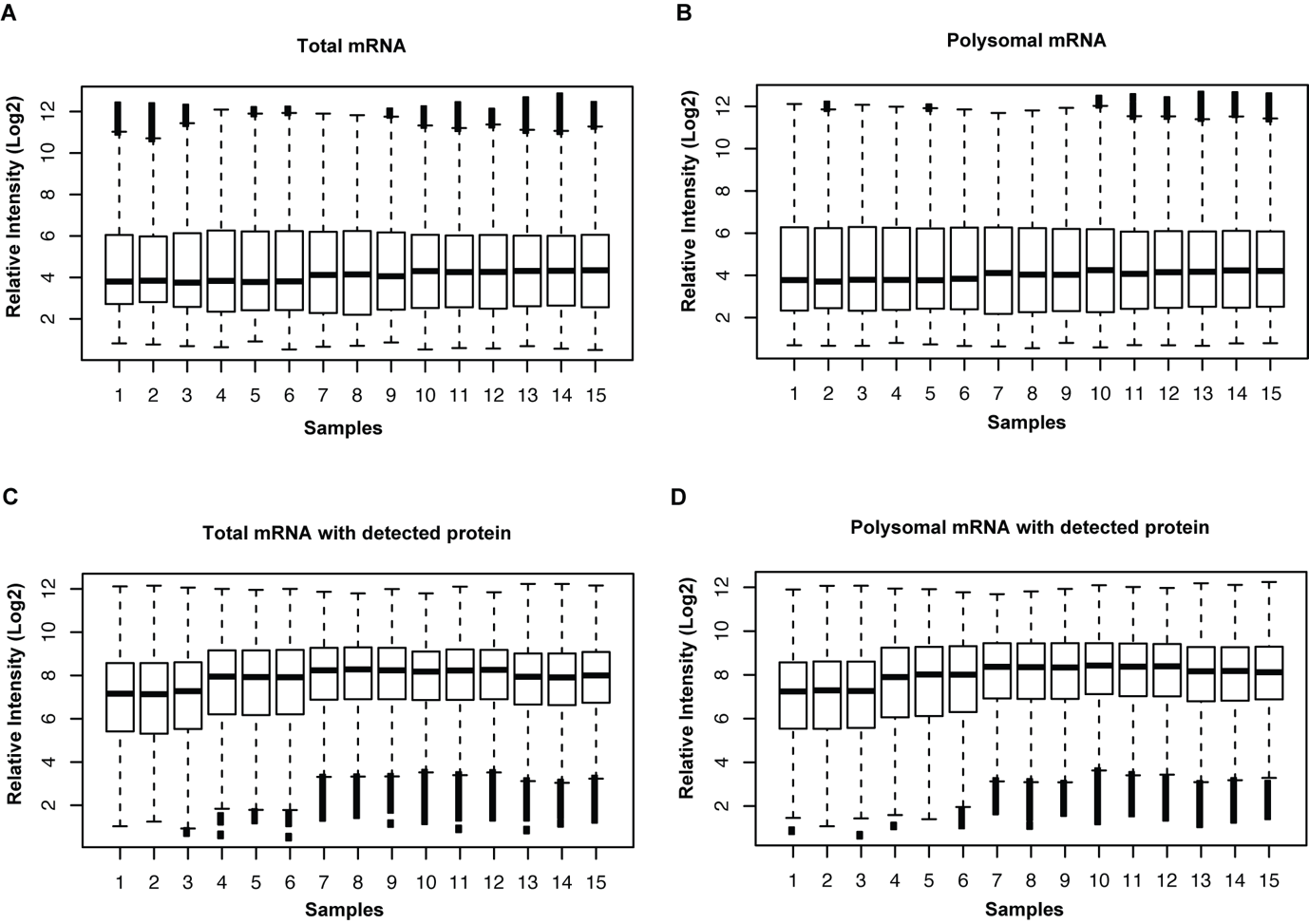


Figure S1. Expression bias of detected protein coding genes and the predicted subcellular localization. Boxplots of the total mRNA (A) and polysomal mRNA (B) identified. (C) Boxplot of the total mRNA (C) and polysomal mRNA (D) of the subset for which proteins have been identified.


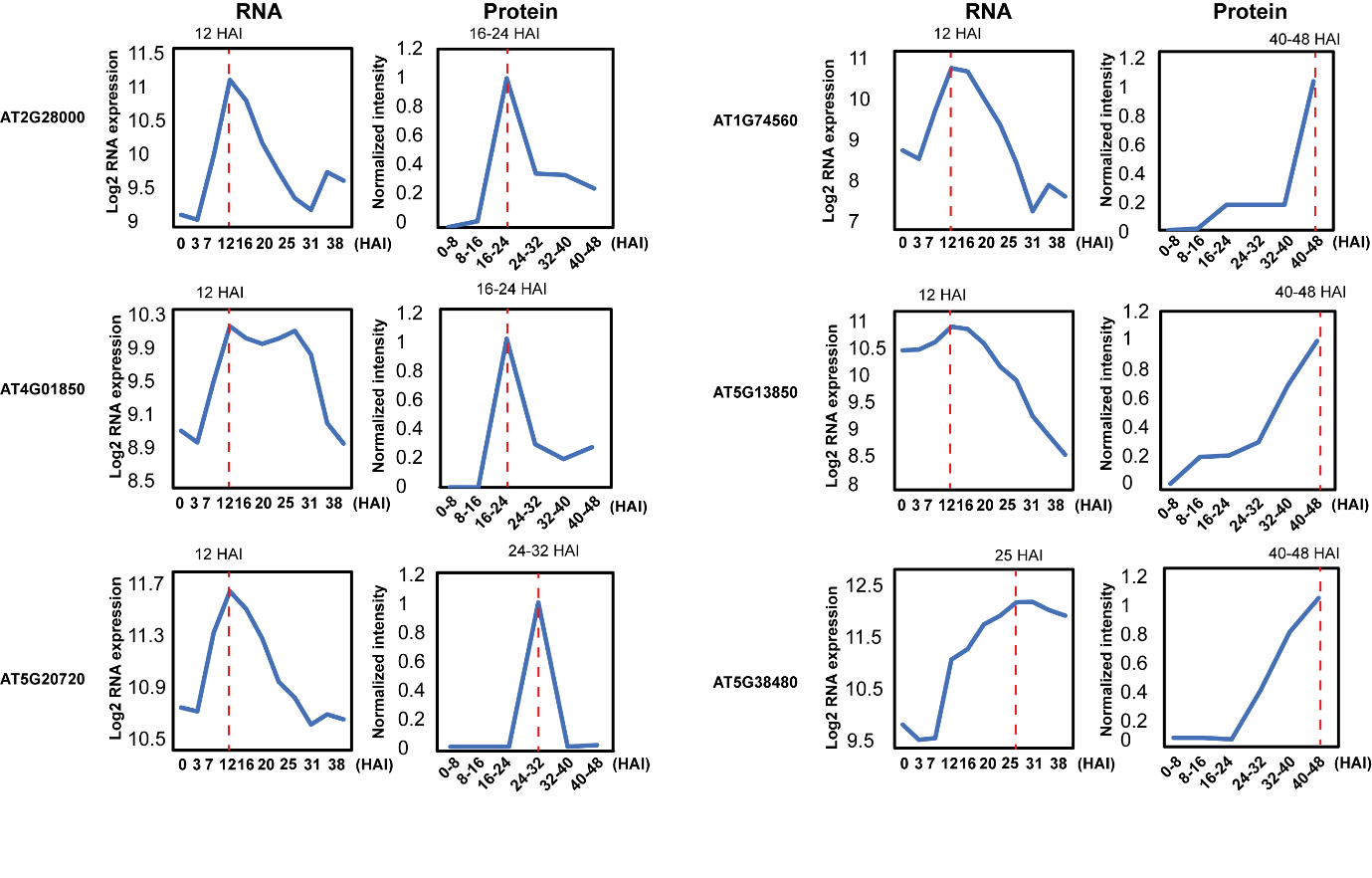


Figure S2. Delayed gene validation. Gene candidates identified as DUGs in current study are validated using two public data sets. The transcriptomic data by Dekkers *et al.*, 2013 represents transcriptome data measured at different time-points during seed germination (0-38hours after imbibition (HAI)) and the set of (Galland *et al.*, 2014) neosynthesized protein identified at 0-48 HAI. The time point for the peak of the transcript level and the protein synthesis signal during seed germination are labelled with red dash lines.


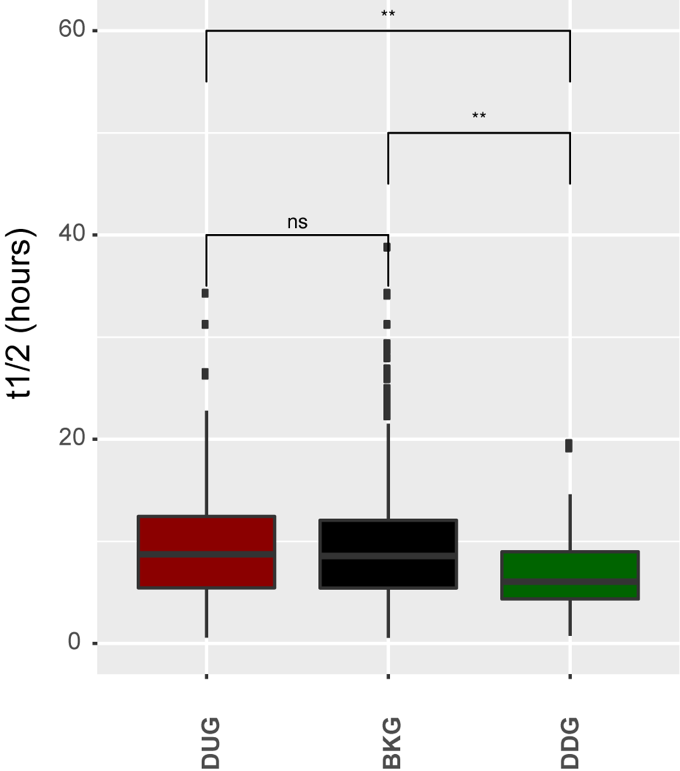


Figure S3 (A) mRNA half-life of the protein delayed genes. mRNA half-life (hours) identified in the Delayed Response Up-regulated Genes (DUG) and the Delayed Response Down-regulated genes (DDG) sets compared with the background (BKG). (**P < 0.01, ns is not significant, Wilcoxon rank-sum test).


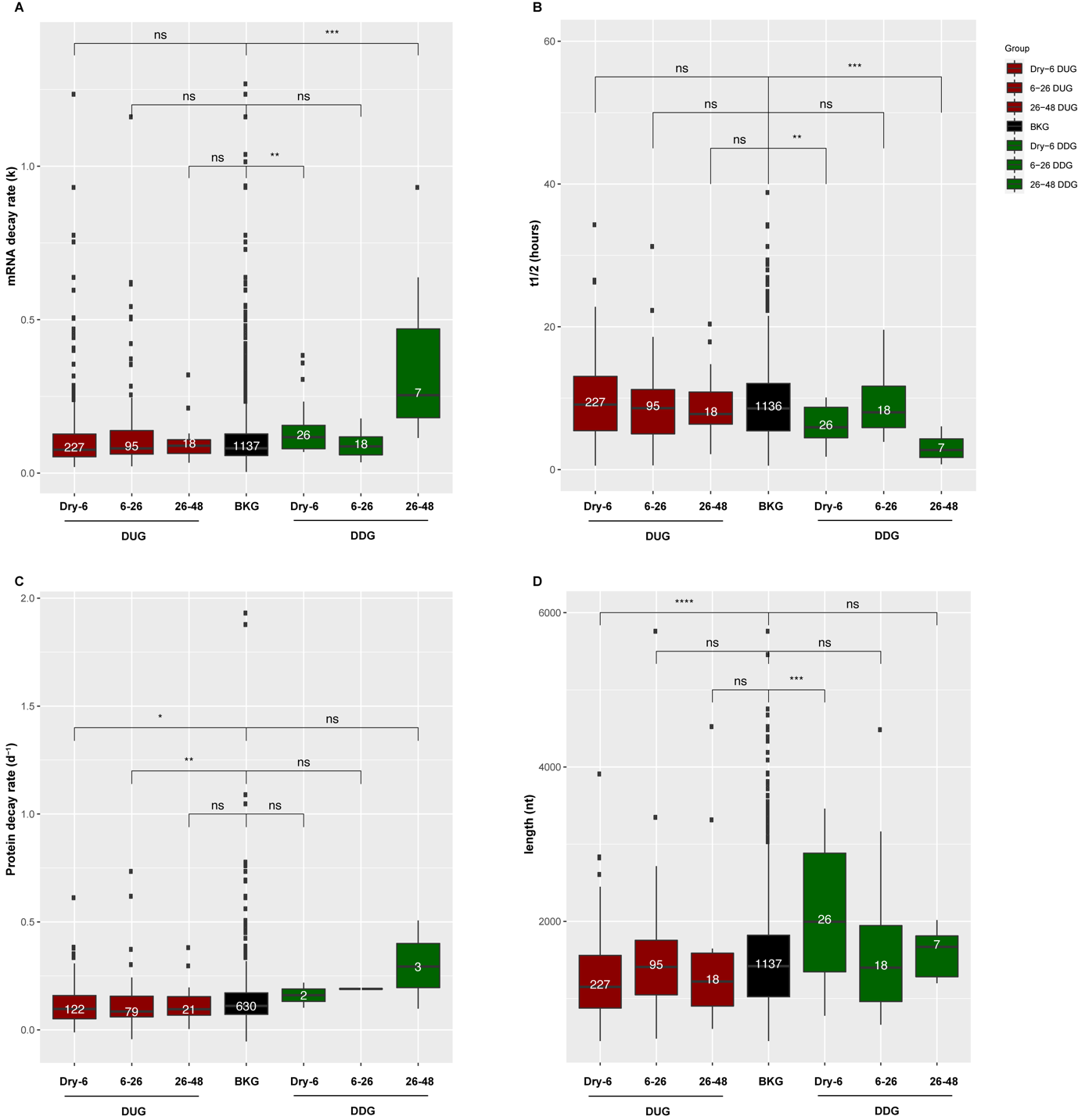


Figure S4 mRNA decay rate, half-life, protein decay rate and mRNA length of the Delayed Response genes grouped by the stages during seed germination. (A) mRNA decay rate and (B) half-life (hours), (C) protein decay rate (days) and (D) mRNA length (nt) of the Delayed Response Up-regulated Genes (DUG) and the Delayed Response Down-regulated genes (DDG) sets compared with the background (BKG). The groups represent the germination stages at which the delay occurs. The number of gene in each group are indicated (****P < 0.0001, ***P < 0.001, **P < 0.01, *P < 0.05, ns is not significant, Wilcoxon rank-sum test).

References

**Dekkers, B.J., Pearce, S., van Bolderen-Veldkamp, R.P., Marshall, A., Widera, P., Gilbert, J., Drost, H.G., Bassel, G.W., Muller, K., King, J.R., Wood, A.T., Grosse, I., Quint, M., Krasnogor, N., Leubner-Metzger, G., Holdsworth, M.J. and Bentsink, L.** (2013) Transcriptional dynamics of two seed compartments with opposing roles in Arabidopsis seed germination. *Plant Physiol*, **163**, 205-215.

**Galland, M., Huguet, R., Arc, E., Cueff, G., Job, D. and Rajjou, L.** (2014) Dynamic proteomics emphasizes the importance of selective mRNA translation and protein turnover during Arabidopsis seed germination. *Mol Cell Proteomics*, **13**, 252-268.
